# Safety of ready‐to‐eat chicken in Burkina Faso: Microbiological quality, antibiotic resistance, and virulence genes in *Escherichia coli* isolated from chicken samples of Ouagadougou

**DOI:** 10.1002/fsn3.650

**Published:** 2018-04-16

**Authors:** Namwin S. Somda, Ouindgueta J. I. Bonkoungou, Cheikna Zongo, Assèta Kagambèga, Imael H. N. Bassolé, Yves Traoré, Jacques Mahillon, Marie‐Louise Scippo, Joseph D. Hounhouigan, Aly Savadogo

**Affiliations:** ^1^ Laboratoire de Biochimie et d'Immunologie Appliquée (LABIA) Unité de Formation et de la Recherche en Sciences de la Vie et de la Terre Ecole Doctorale Sciences et Technologies Université Ouaga I Pr Joseph Ki‐Zerbo Ouagadougou Burkina Faso; ^2^ Laboratoire National de Santé Publique (LNSP) Ouagadougou Burkina Faso; ^3^ Laboratoire de Biologie Moléculaire d'Epidémiologie et de Surveillance des agents Transmissibles par les Aliments (LABESTA) Unité de Formation et de la Recherche en Sciences de la Vie et de la Terre Ecole Doctorale Sciences et Technologies Université Ouaga I Pr Joseph Ki‐Zerbo Ouagadougou Burkina Faso; ^4^ Faculté d'ingénierie Biologique Agronomique et Environnementale Université catholique de Louvain Louvain‐la‐Neuve Belgium; ^5^ Département des Sciences des Denrées alimentaires Centre de recherche FARAH—Secteur Santé Publique Vétérinaire Université de Liège Liège Belgium; ^6^ Laboratoire de Biochimie Microbienne et de Biotechnologie Alimentaires Université d'Abomey Calavi Abomey Calavi Benin

**Keywords:** antimicrobial resistance, Burkina Faso, diarrheagenic *E. coli*, grilled/flamed chickens, hygienic quality

## Abstract

In Burkina Faso, flamed/grilled chickens are very popular and well known to consumers. The aim of this study was to evaluate the microbiological quality, the antibiotic resistance, and the virulence gene from *Escherichia coli* isolated from these chickens in Ouagadougou. A total of 102 grilled, flamed, and fumed chickens were collected in Ouagadougou and analyzed, using standard microbiological methods. All *E. coli* isolates were checked with the antimicrobial test and also typed by 16‐plex PCR. The mean of aerobic mesophilic bacteria (AMB) and thermo‐tolerant coliforms (TTC) was found respectively between 6.90 ± 0.12 × 10^7^ CFU/g to 2.76 ± 0.44 × 10^8^ CFU/g and 2.4 ± 0.82 × 10^7^ CFU/g to 1.27 ± 0.9 × 10^8^ CFU/g. *E. coli* strains were found to 27.45%. Forty samples (38.24%) were unacceptable based on the AMB load. Fifty‐nine samples (57.85%) were contaminated with TTCs. Low resistance was observed with antibiotics of betalactamin family. Diarrheagenic *E. coli* strains were detected in 21.43% of all samples. This study showed that flamed/grilled chickens sold in Ouagadougou could pose health risks for the consumers. Need of hygienic practices or system and good manufacturing practices is necessary to improve the hygienic quality of flamed/grilled chickens. Our results highlight the need of control of good hygiene and production practices to contribute to the improvement of the safety of the products and also to avoid antibiotic resistance. Slaughter, scalding, evisceration, plucking, bleeding, washing, rinsing, preserving, grilling, and selling may be the ways of contamination.

## 1 INTRODUCTION

Microbial food safety is an increasing public health concern worldwide. According to the World Health Organization (WHO), an important proportion of diarrhea in the world is of food origin (WHO 2013). More than 219,000 people die yearly in the world because of gastroenteritis due to lack of hygiene. Developing countries are the most affected by include cholera, campylobacteriosis, and infections to *Escherichia coli*, shigellosis, brucellosis, hepatitis A, and salmonellosis. Among the most appreciated street foods, meat products occupied a very good position. However, the poor sanitary practices during cooking and sale multiply the risk of microbial contamination. High ambient temperatures especially in tropical environments have been described as the major factors responsible for facilitating the access and multiplication of bacterial contaminants in meat products (Barro et al., 2006; Mankee et al., 2003). *E. coli* which is the first germ of fecal contamination indicators are responsible for several infections. In developing countries, the majority of the population treat these infections by self‐medication because of ignorance and refusal of cure. This practice is one of the causes of the emergence of antibiotic‐resistant bacteria (Rubin & Samore, 2002). In addition, the uncontrolled use of antibiotics in the livestock sector for animal diseases treatment and prevention as well as a growth promoters contribute to increase the spreading of antibiotic‐resistant bacteria (Kagambèga et al., 2013). Antimicrobials are valuable means to treat clinical diseases and keep healthy and growth promotion. However, the treatment of all herds and flocks with antimicrobials for increasing the growth and preventing illness has become an endless debate (Witte, 1998). Often whole flocks or herds of sick animals are treated at once, containing animals that are not sick. It is now generally accepted that the main risk factor for the increase in resistance to pathogenic bacteria is the anarchical use of antibiotics (Somda et al., 2017).

In Burkina Faso, more than 50,000 poultry are transported, killed, and consumed every day in Ouagadougou (DSMRA 2015). The hygiene often fails during slaughtering, scalding, evisceration, plucking, bleeding, washing, and rinsing, and increase the health risk associated with the consumption of this meat (Coulibaly, Bakayoko, & Karou, 2010). The aims of this study were to assess (1) microbiological quality of ready‐to‐eat chicken borne, (2) antibiotic resistance, and virulence genes of *E. coli* isolated in these chickens.

## 2 MATERIAL AND METHODS

### 2.1 Samples collection

From September to December 2016, 102 chickens meats were collected from 102 producers in 59 sectors distributed on three crowns in Ouagadougou. These samples were composed of 66 grilled chickens (nine in crown 1, 28 in crown 2, and 27 in crown 3), 29 flamed chickens (two in crown 1, 18 in crown 2, and nine in crown 3), four fumed chickens (two in crown 1, two in crown 2, and 0 in crown 3), and three chickens prepared around fire (three in crown 1, 0 in crown 2, and 0 in crown 3). The samples were transported to the laboratory and kept at 4°C. The microbiological examination was started within the eight following hours.

All microbiological and molecular tests were carried out at *Laboratoire National de Santé Pulique (LNSP)* Ouagadougou, Burkina Faso.

### 2.2 Microbiological analysis

Twenty‐five grams of each meat sample (for chicken a mixture of meat from neck, breast, wings, and legs) was added into 225 ml of Buffered Peptone Water (Liofilchem, Teramo, Italy) and homogenized using a stomacher (400 Circulator; Seward, London, UK). Dilutions of 10 to 10 were realized from the stock solution according to ISO methods (ISO6887‐2 2004).

### 2.3 Aerobic mesophilic bacteria's enumeration

These microorganisms were enumerated on the plat count agar (PCA) medium according to the standard (ISO4833 2003). Briefly, 1 ml of each dilution was plated on PCA and incubated at 30°C for 72 ± 3 hr. The enumeration of the colonies was carried out on two successive dilution dishes (less than 300 colonies and more than 10 colonies on a dish). The results were expressed according to the standard (NFV08‐102 1998) using the following formula:

(1) $$N=\frac{\Sigma \;c}{V\;(n1+0.1\;n2)d}\left(\text{CFU/g}\right)$$

Σc = the total of colonies counted on all the retained dishes of two successive dilutions and of which at least one dish contains 10 colonies; V = volume of inoculum applied to each dish in milliliters; n1 = dish number retained at the first dilution; n2 = dish number retained at the second dilution; 0.1 = dilution factor; d = dilution ratio corresponding to the first dilution retained.All the results were interpreted according to the standard (AFSSA 2007‐SA‐0174 2008). The threshold of detection of this germ was 10^8^ CFU/g.

### 2.4 Thermo‐tolerant coliforms (*E. coli*) enumeration

Seeding was carried out in a double layer on violet red bile lactose agar and incubated at 44°C for 24 hr according to standard (NFV08‐053 2002). Bacteria were enumerated according to the formula described above. The threshold of detection of this germ was 10^2^ CFU/g (AFSSA 2007‐SA‐0174 2008). After enumeration, two or three colonies were subcultured on Bromocresol purple agar and eosin methylene blue agar and incubated at 37°C for 24 hr. *E. coli* were characterized using API 20E system and isolated strains preserved at −30°C for antibiotic resistance test and molecular characterization.

### 2.5 Coagulase‐positive staphylococci enumeration

About 0.1 ml of the suspension was streaked on to the Baird Parker agar with egg yolk with potassium tellurite and carefully spread with a spreader (ISO6888‐2/A1 2003). Incubation was carried out at 37°C for 24–48 hr. All the dishes where there was growth of small black, shiny colonies surrounded by a transparent halo and an opaque border of 2–5 mm in diameter were retained to enumerate. The enumeration was carried out according to the formula described above. The threshold of detection of this germ was 10^3^ CFU/g according to AFSSA 2007‐SA‐0174 (2008).

### 2.6 *Salmonella* characterization

*Salmonella* was tested for xylose lysine desoxycholate and *Salmonella–Shigella* (SS) agar medium according to ISO6579 (2002). The threshold of detection of this germ was 0 germ/25 g (AFSSA 2007‐SA‐0174 2008).

### 2.7 Antibiotic resistance test

All isolates were also tested for susceptibility to 14 different antimicrobial agents using the disk diffusion method on Mueller Hinton II agar (Bio‐Rad France), following the European Committee on Antimicrobial Susceptibility Testing (EUCAST) guidelines (EUCAST 2013). *E. coli* ATCC 25922 and ATCC 35218 were used as a control. The antimicrobial disks (Himedia, India) used were nalidixic acid (30 μg), ciprofloxacin (5 μg), ampicillin (10 μg), amoxicillin (25 μg), cefotaxime (30 μg), imipenem (10 μg), tetracycline (30 μg), gentamicin (10 μg), chloramphenicol (30 μg), ceftriaxone (30 μg), norfloxacin (10 μg), ticarcillin (75 μg), amoxicillin/clavulanic acid (30 μg), and sulfamethoxazole/trimethoprim (25 μg). Inhibition diameters of the antibiotics were interpreted according to the European Committee on Antimicrobial Susceptibility Instructions (EUCAST 2013). The multiresistance is defined as the resistance to at least three different antibiotics families (Magiorakos et al., 2011).

### 2.8 16‐plex PCR assay

The presence of STEC, EPEC, ETEC, EIEC, and EAEC on grilled chicken meat samples was detected by 16‐plex PCR for the genes *uidA, pic, bfp, invE, hlyA, elt, ent, escV, eaeA, ipaH, aggR, stx1, stx2, estIa, estIb,* and *ast*. The primers and PCR conditions were as previously described (Antikainen et al., 2009). The nucleotide sequences and predicted sizes of the amplified products for the specific oligonucleotide primers used in this study are shown in Table 1. The following criteria for identification of *E. coli* pathogroups were used: for STEC, the presence of *stx1* and/or *stx2* and possibly *eaeA, escV, ent,* and EHEC‐*hly;* for EPEC, the presence of *eaeA* and possibly *escV, ent,* and *bfpB* (the absence of *bfpB* indicated a EPEC); for ETEC, the presence of *elt* and/or *estIa* or *estIb*; for EIEC, the presence of *invE* and *ipaH*; and for EAEC, the presence of *pic* and/or *aggR*. For DNA extraction, a loopful of bacterial growth was taken from the first streaking area of the plate. It was suspended in 250 μl of sterile water in an Eppendorf tube, boiled at 100°C for 10 min and centrifuged.

**Table 1:** Oligonucleotide primers used for detection of the virulence genes

Pathogroups	Genes	Sequences	bp	Concentration	References
STEC, EPEC	*eae‐*F	TCAATGCAGTTCCGTTATCAGTT	482	0.1	Vidal et al. (2005)
	*eae‐*R	GTAAAGTCCGTTACCCCAACCTG			
	*escV‐*F	ATTCTGGCTCTCTTCTTCTTTATGGCTG	544	0.4	Müller et al. (2007)
	*escV‐*R	CGTCCCCTTTTACAAACTTCATCGC			
	*ent‐*F	TGGGCTAAAAGAAGACACACTG	629	0.4	Müller et al. (2007)
	*ent‐*R	CAAGCATCCTGATTATCTCACC			
Typical EPEC	*bfpB‐*F	GACACCTCATTGCTGAAGTCG	910	0.1	Müller et al. (2007)
	*bfpB‐*R	CCAGAACACCTCCGTTATGC			
STEC	*hlyEHEC‐*F	TTCTGGGAAACAGTGACGCACATA	688	0.1	Antikainen et al. (2009)
	*hlyEHEC‐*R	TCACCGATCTTCTCATCCCAATG			
	*stx1A‐*F	CGATGTTACGGTTTGTTACTGTGACAGC	244	0.2	Müller et al. (2007)
	*stx1A‐*R	AATGCCACGCTTCCCAGAATTG			
	*stx2A‐*F	GTTTTGACCATCTTCGTCTGATTATTGAG	324	0.4	Müller et al. (2007)
	*stx2A‐*R	AGCGTAAGGCTTCTGCTGTGAC			
EIEC	*ipaH‐*F	GAAAACCCTCCTGGTCCATCAGG	437	0.1	Antikainen et al. (2009)
	*ipaH‐*R	GCCGGTCAGCCACCCTCTGAGAGTAC		0.1	Brandal et al. (2007)
	*invE‐*F	CGATAGATGGCGAGAAATTATATCCCG	766	0.2	Müller et al. (2007)
	*invE‐*R	CGATCAAGAATCCCTAACAGAAGAATCAC			
EAEC	*aggR‐*F	ACGCAGAGTTGCCTGATAAAG	400	0.2	Müller et al. (2007)
	*aggR‐*R	AATACAGAATCGTCAGCATCAGC			
	*pic‐*F	AGCCGTTTCCGCAGAAGCC	1,111	0.2	Müller et al. (2007)
	*pic‐*R	AAATGTCAGTGAACCGACGATTGG			
	*astA‐*F	TGCCATCAACACAGTATATCCG	102	0.4	Müller et al. (2007)
	*astA‐*R	ACGGCTTTGTAGTCCTTCCAT			
ETEC	*LT‐*F	GAACAGGAGGTTTCTGCGTTAGGTG	655	0.1	Müller et al. (2007)
	*LT‐*R	CTTTCAATGGCTTTTTTTTGGGAGTC			
	*STIa‐*F	CCTCTTTTAGYCAGACARCTGAATCASTTG	157	0.4	Müller et al. (2007)
	*STIa‐*R	CAGGCAGGATTACAACAAAGTTCACAG			
	*STI‐*F	TGTCTTTTTCACCTTTCGCTC	171	0.2	Müller et al. (2007)
	*STI‐*R	CGGTACAAGCAGGATTACAACAC			
*E. coli*	*uidA‐*F	ATGCCAGTCCAGCGTTTTTGC	1,487	0.2	Müller et al. (2007)
	*uidA‐*R	AAAGTGTGGGTCAATAATCAGGAAGTG			

STEC, Shiga toxin‐producing *E. coli*; EPEC, enteropathogenic *E. coli*; EIEC, enteroinvasive *E. coli*; EAEC, enteroaggregative *E. coli*; ETEC, enterotoxigenic *E. coli*.

PCR was performed in a reaction of 20 μl containing 2.5 μl 10× PCR buffer (Solis Biodyne), 0.75 μl dNTPs (10 mmol/L), 0.25 μl MgCl_2_ (50 mmol/L), 0.2 μl Taq DNA polymerase (5 U/μl), and 0.5 μl of each mixture of the 16 primer pairs at the concentrations listed in Table 1; 12.8 μl of PCR‐grade water and 2.5 μl of DNA sample were added to bring the final volume to 10 μl. The cycling conditions used in the thermal cycler (Applied Biosystem, 2720 thermal cycler, Singapore) were 98°C for 30 s, 35 cycles of 98°C for 30 s, 63°C for 60 s, and 72°C for 90 s with a final extension at 72°C for 10 min.

The amplified PCR products were separated by agarose gel (1.5% w/v) electrophoresis and visualized under UV light (Bioblock Scientific, Illkirch, CEDEX) after staining with ethidium bromide. Reference strains RHE 4283 (E 2348/69) for EPEC, FE94725 (Burkina Faso, beef) for ETEC, FE102301 (Burkina Faso, beef) for STEC, RHE 6647 (145‐46‐215, Statens Serum Institut [SSI], Copenhagen, Denmark) for EIEC, IH 56822 (patient isolate (Keskimäki, Mattila, Peltola, & Siitonen, 2000), for EAEC, and FE95562 (Burkina Faso, beef) for STEC‐ETEC were included in each PCR run. All the 16‐plex PCR positive results were confirmed by single PCRs.

## 3 RESULTS

### 3.1 Microbiological analysis

The average loads of various microorganisms determined are summarized in Table 2. The average loads of aerobic mesophilic bacteria (AMB) and thermo‐tolerant coliforms (TTCs; including *E. coli*) varied respectively between 6.90 ± 0.12 × 10^7^ CFU/g to 2.76 ± 0.44 × 10^8^ CFU/g and 2.4 ± 0.82 × 10^7^ CFU/g to 1.27 ± 0.9 × 10^8^ CFU/g. No *Salmonella* ssp. and *Staphylococcus*‐positive coagulase were found. According to the AFSSA 2007‐SA‐0174 standard referring to the load of the AMB, about 38.24% (40/102, including 25 grilled chickens, 11 flamed chickens, and four fumed chickens) of the analyzed samples were unacceptable (superior to 10^8^ CFU/g) (Table 3). About 57.85% (59/102, including 36 grilled chicken, 16 flamed chickens, four fumed chickens, and three chickens around the fire) of the samples were unacceptable (superior to 10^2^ CFU/g) according to their load of TTCs (Table 3). *E. coli* were found in 27.45% (28/102 including 20 in grilled chickens, seven in broiler chickens, and one in fumed chicken) of the samples analyzed, as shown in Table 3. In addition to *E. coli*, others pathogens including *Klebsiella* spp. (in one sample), *Pantoea* spp. (in eight samples), *Serratia ficaria* (in two samples), and *Kluyera* spp. (in two samples) were isolated in 12.75% (13/102 samples). Six were contaminated with both *E. coli* and others pathogens, while in single‐sample chickens, one was both contaminated with *E. coli* and *Pantoea* spp.

**Table 2:** The average loads of the various microorganisms

Products	Grilled chicken (*n* = 66)	Flamed chicken (*n* = 29)	Fumed chicken (*n* = 4)	Chickens around the fire (*n *= 3)	Total (*n* = 102)
AMB (10^8^ CFU/g)	1.03 ± 1.36	1.09 ± 1.36	2.76 ± 0.44	0.690 ± 0.115	1.12 ± 1.4
TTC (10^8^ CFU/g)	0.24 ± 0.82	0.28 ± 0.86	1.27 ± 0.9	0.934 ± 1.61	0.32 ± 0.87

AMB, aerobic mesophilic bacteria; TTC, thermo‐tolerant coliforms; CFU/g, colony‐forming unit per gram; *n*, number.

**Table 3:** Contamination frequency of different chickens analyzed

Products	Grilled chicken *n* = 66 (%)	Flamed chicken *n* = 29 (%)	Fumed chicken *n* = 4 (%)	Chickens around the fire *n* = 3 (%)	Total *n* = 102 (%)
AMB > 10^8 ^CFU/g	25 (38)	11 (38)	4 (100)	0	40 (38.24)
TTC	36 (55)	16 (55)	4 (100)	3 (100)	59 (57.85)
*E. coli*	20 (30)	7 (24)	1 (25)	0	28 (27.45)
*Salmonella* spp.	0	0	0	0	0
*Staphylococcus aureus*	0	0	0	0	0
Others bacteria	10 (15)	3 (10)	0	0	13 (12.75)
*E. coli* + others bacteria	6 (9)	1 (3)	0	0	7 (6.86)

AMB, aerobic mesophilic bacteria; TTC, thermo‐tolerant coliforms; *E. coli*,* Escherichia coli*; CFU/g, colony‐forming unit per gram; *n*, number; %, percentage.

Others bacteria: *Enterobacter sakazakii*,* Klebsiella pneumoniae*,* Klyvera* spp., *Pantoea* spp., *Serratia ficaria*.

### 3.2 Antimicrobial susceptibility testing of griller/flamed chickens isolates

**Figure 1:**
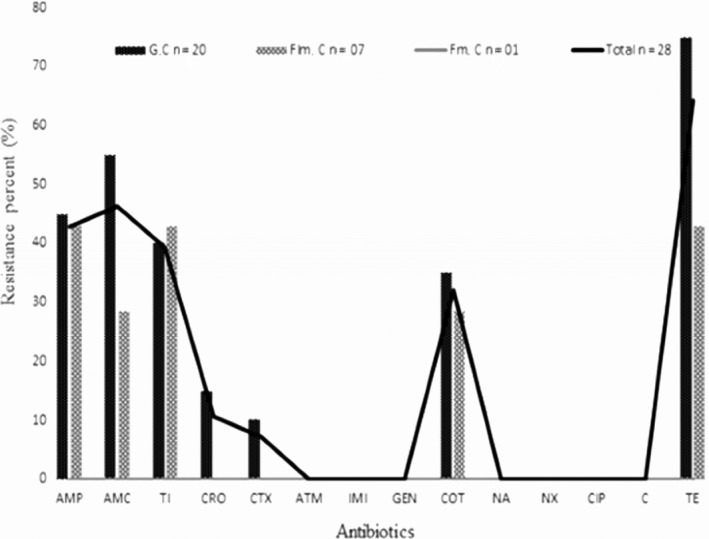
Antimicrobial resistance of *Escherichia coli* strains isolated from grilled/flamed chicken meat. AMP, ampicillin; ATM, aztreonam; AMC, amoxicillin/clavulanate; CRO, ceftriaxone; CTX, cefotaxime; NX, norfloxacin; COT, trimethoprim/sulfamethoxazole; C, chloramphenicol; CIP, ciprofloxacin; GEN, gentamicin; IMI, imipenem; NA, nalidixic acid; TE, tetracycline; TC, ticarcillin; %, percentage; *n*, number; G.C, grilled chicken; Flm.C, flamed chicken; Fm.C, fumed chicken

Globally, the *E. coli* isolated from fumed, grilled, and flamed chickens showed a low resistance to antibiotic teste. All isolates were sensitive to imipenem, ciprofloxacin, nalidixic acid, norfloxacin, gentamicin, aztreonam, and chloramphenicol. However, resistance was observed with cefotaxime (7.14%), ceftriaxone (10.71%), sulfamethoxazole/trimethoprim (32.14%), ticarcillin (39.3%), amoxicillin/clavulanic acid (46.43%), ampicillin (42.86%), and tetracycline (64.3%) (Figure 1). Twenty‐one percent (6/28) of tested strains were extended‐spectrum‐beta‐lactam (ESBL) positives.

### 3.3 Diarrheagenic *E. coli* characterization

**Figure 2:**
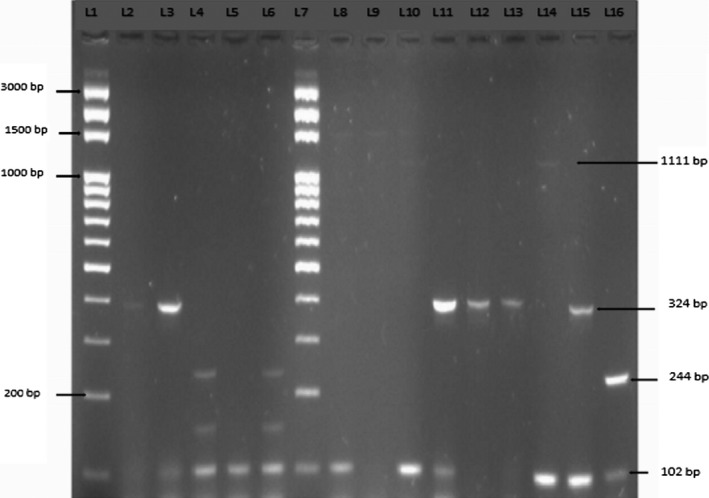
Example of the 16‐plex PCR results to DEC isolated from grilled/flamed chicken. L1 = marker, L2 = RHE6647 (EIEC), L3 = IHE56822 (EAEC), L4 = FE102301 (STEC), L5 = FE94725 (ETEC), L6 = FE95562 (STEC‐ETEC), L7 = marker, L8 = G.C_2_, L9 = G.C_6_, L10 = G.C_9_, L11 = G.C_12_, L12 = G.C_34_, L13 = G.C_38_, L14 = G.C_60_, L15 = Flm.C_27_, L16 = Flm.C_28_

16‐plex PCR was used to detect virulence genes carried by diarrheagenic *E. coli* and to classify the strains as STEC, EPEC, ETEC, EIEC, or EAEC. Diarrheagenic *E. coli* were detected in 21.43% (6/28) of all samples. Only *STIa, stx2A, invE, astA,* and *aggR* virulence genes were detected (Table 4). The six detected diarrheagenic *E. coli* were as follows: EAEC and ETEC (in two samples each) and STEC and EIEC (in one sample each) (Figure 2). No EPEC was detected. According to the nature of samples (grilled chickens, flamed chickens, and fumed chickens), prevalence of diarrheagenic *Escherichia coli* (DEC) was 20% (4/20), 28.57% (2/7) from grilled chickens and flamed chickens, respectively.

**Table 4:** Virulence genes detected by 16‐plex PCR of *Escherichia coli* strains isolated in grilled/flamed chicken meat

DEC pathovars	Virulence genes	Grilled chicken (*n* = 20)	Flamed chicken (*n* = 7)	Fumed chicken (*n* = 1)
STEC (*n* = 1)	*Stx2A*	+	–	–
EAEC (*n* = 2)	*asta, aggR*	–	+	–
EIEC (*n* = 1)	*invE*	+	–	–
ETEC (*n* = 2)	*STIa*	+	–	–
Total *n* = 6 (%)		4 (20)	2 (28.57)	0

DEC, Diarrheagenic *Escherichia coli*;* n*, number; %, percent; +, presence; –, absence.

## 4 DISCUSSION

According to Food and Agriculture Organization of the United Nations (FAO), the risk of food poisoning associated with street food remains a threat in many parts of the world, with microbiological contamination being one of the major problems. It is recognized that food‐borne pathogens represent a serious health hazard, with the risk mainly depending on the type of food and the way of cooking and conservation. The ignorance of street sellers as the cause of food‐borne illnesses is a risk factor that cannot be ignored (FAO 2007). To our knowledge, this is the first study about *E. coli* in flamed, grilled, and fumed chicken meat. The results of the microbiological analyses of flamed, grilled, and fumed chicken meat showed the highest load in both AMB (threshold value for contamination superior to 10^8 ^CFU/g) and TTCs. *E. coli* are the first germs of fecal contamination indicators. The presence of *E. coli* and AMB is an indicator of lack of hygiene in production process. As the chickens are flamed or grilled, some questions like the route of contamination remain and need to be addressed. This hygienic issue may be due to the possible contamination of chickens since the abattoir, and/or during the process of chickens grilling and/or postprocessing contamination due to unhygienic sales environment and handling of grilled chickens. Consequently, the consumption of these contaminated products could be a public health risk for consumers. Previous studies in West Africa have showed contamination of fresh chicken meat and offals by AMBs and TTCs (Attien et al., 2013; Ilboudo, Savadogo, Samandoulougou, & Abre, 2016). Otherwise, these studies have shown that the conditions for slaughter, scalding, evisceration, plucking, bleeding, washing, rinsing, preserving, grilling, and selling may be the ways of contamination. These failures of the hygiene rules are generally the cause of the contamination of fresh meat. The contamination could be also at the level of the sale material, the seller himself, and also the quality of the grilling (insufficient time of grilling) as stated some researchers (Khallaf et al., 2014). Our survey results showed that during the grilling process, some others sellers used dirty water to reduce fire intensity when it is high. The charcoal used for grilling could also be the source of the contamination because the conditions of production of the charcoal do not obey the rules of hygiene. In addition, others sellers were alone and were at the same time responsible for the whole chain of meat production. They slaughtered the chickens, scalded them, eviscerated them, plucked them, washed them, preserved them, burned them, and even received the money. These practices could increase the risk of contamination of meat at the end of production. Barro et al. (2006) and Gedik, Voss, and Voss (2013) showed that money handling constitutes another risk factor of street foods contamination. Money can get contaminated and may thus play a role in the transmission of microorganisms to other people, during the selling.

No *Salmonella* and *Staphylococcus* coagulase positive were found in this study. However, others studies notified the presence of these bacteria in fresh poultry meat (Attien et al., 2013; Kagambèga et al., 2013; Khallaf et al., 2014; Nzouankeu, Ngandjio, Ejenguele, Njine, & Wouafo, 2010).

Our results showed that the great majority of *E. coli* isolates from grilled/fumed chicken were susceptible to ciprofloxacin, chloramphenicol, norfloxacin, nalidixic acid, aztreonam, imipenem, and gentamicin. However, resistance was observed to antibiotics of betalactamin family such as ampicillin, amoxicillin/clavulanic acid, ceftriaxone, ticarcillin cefotaxime, and others families such as tetracycline and trimethoprim/sulfamethoxazole. ESBL was found in six strains. Antimicrobials are used in the absence of illness to prevent diseases when animals are susceptible to infection (Turtura, Massa, & Ghazvinizadeh, 1990). This practice is very usual in developing countries where outbreak is caused by enteric pathogens which are the sources of farms poultry diseases. Our results were in agreement with previous studies realized on fresh and broiler meat chickens in Canada, Spain, in Iran (Cortés et al., 2010; Vincent et al., 2010). In slaughterhouse, resistant strains from the gastrointestinal tract may infect chicken carcasses and, as a result, chicken meats are often related to multiresistant *E. coli*. Also, eggs become infected during laying (Reza, Mehdi, Faham, & Mohammad, 2014). Therefore, antimicrobial‐resistant fecal *E. coli* from poultry can infect humans directly and indirectly with food. Though seldom, these resistant bacteria may colonize in the human gastrointestinal tract and may also transfer resistance bacteria to human endogenous flora (Reza et al., 2014).

In Burkina Faso, chicken meat is very popular, but production conditions are very poor and could lead to diarrheal diseases to consumers. The present study was the first conducted in Burkina Faso to detect diarrheagenic *E. coli* based on the presence of the virulence genes in bacterial cultures derived from grilled/flamed chickens. In 2012, a similar study was conducted by Kagambega et al. (2012) in beef, mutton, and fresh chicken meat. They showed that EPEC, ETEC, and EAEC were detected less often than STEC in beef and mouton meat and intestine samples, except in chickens, which seem to be the major carriers of atypical EPEC. However, the present study carried out on grilled/flamed chickens showed that ETEC and EAEC were detected each on two strains but STEC and EIEC were detected each on one strain. No EPEC is detected in our study. However, EPEC was detected in similar studies conducted in others countries (Farooq, Hussain, Mir, Bhat, & Wani, 2009; Nzouankeu et al., 2010). *E. coli* strains as STEC can be transmitted via the fecal‐oral route and contamination often occurs through the ingestion of contaminated food or water, direct contact with animals, interhumans, or contaminated objects, or more rarely by inhalation (Crump et al., 2002; Grant et al., 2008). Presence of the DEC in grilled/flamed chicken meat refect poor food hygiene and the common occurence of potential pathogens from human in Burkina Faso. Hygiene rules must be applied strictly to the slaughterhouse and in the sale outlets to avoid contamination of grilled/flamed meat by DEC.

Our study showed that most of our samples analyzed contain amounts of germs that exceed the acceptable limits to the standards established for both mesophilic aerobic total flora, fecal coliforms, and diarrheagenic *E. coli* pathogroups. It is necessary to set up good hygienic practices in the production of grilled, fumed, and flamed chickens to ensure the quality of finished products. In addition, intervention strategies, such as promoting hand washing with soap and good hygienic practices at the slaughterhouses and sales outlets, can have a sound practical impact on public health.

## 5 CONCLUSION

This study shows also the emergence of betalactamin resistance to *E. coli* isolates in grilled, fumed, and flamed chickens. The resistance of *E. coli* to betalactamins highlights the need for the establishment of a network and continuous monitoring of antibiotic resistance.

## CONFLICT OF INTEREST

None.
